# Phenological responses of corn to agricultural mechanization: Evidence from a wheat-corn double cropping system in China

**DOI:** 10.1371/journal.pone.0312812

**Published:** 2024-11-01

**Authors:** Teng Wang, Fujin Yi, Huilin Liu

**Affiliations:** 1 College of Economics and Management, Nanjing Agricultural University, Nanjing, Jiangsu, China; 2 China Academy for Rural Development, School of Public Affairs, Zhejiang University, Hangzhou, Zhejiang, China; Agricultural Sciences and Natural Resources University of Khuzestan, ISLAMIC REPUBLIC OF IRAN

## Abstract

The development of multi-cropping systems is hampered by the seasonal shortage problem of growing two or three crops within a year. Exploring strategies to alleviate phenological constraints in multi-cropping systems is crucial for increasing grain production. Using a county-level panel dataset with detailed crop progress information from China, this study investigates whether mechanized winter wheat harvest can alleviate the phenological constraints of a shorter growing season for subsequent summer corn in a wheat-corn double cropping system. The results show that mechanized winter wheat harvest considerably extends the length of the summer corn growing season. This spillover effect of mechanized winter wheat harvest is particularly evident in plains and hilly areas with larger farm sizes. Further analysis of the mechanism indicates that mechanized winter wheat harvest affects the length of the summer corn growing season by advancing the planting date and delaying the maturity date. These results underscore the importance of advancing agricultural mechanization to enhance food security under limited land resources.

## 1. Introduction

Ensuring sufficient access to food for everyone is an urgent and daunting global challenge, especially in light of limited arable land and water resources. There is growing evidence that improving cropland use intensity through the adoption of multi-cropping systems is a strong option for increasing grain production [1, 2]. A multi-cropping system refers to harvesting two to three crops from the same plot of land within a year. Previous research has demonstrated that shifting from a single-crop system to a double-crop system has the potential to nearly double annual agricultural output without expanding cropland area [3]. Despite its potential, the development of multi-cropping systems in regions with adequate moisture is primarily constrained by the short average growing season, which in turn hinders the practice of double or triple cropping [4, 5]. Therefore, it is imperative to explore strategies that can alleviate the phenological constraints in multi-cropping systems, which are essential for optimizing land resource utilization and ensuring global food security.

Studies in the fields of climate change and agronomy have explored strategies to alleviate phenological constraints in multi-cropping systems. Research on climate change has suggested that global warming extends the potential growing season, providing opportunities to expand double and triple cropping [6–9]. However, warmer temperatures may also result in earlier harvest of many existing crop varieties, potentially reducing final yields unless these crops can adapt to the extended growing season [10]. Agronomy studies have concentrated on the impact of agricultural technologies in adjusting crop phenology [11]. For example, the adoption of conservation tillage, including no-tillage and reduced-tillage, can advance the planting date for the second crop in a multi-cropping system by reducing the operating time between the first crop harvest and the second crop planting [12–14]. Furthermore, techniques such as dry sowing and seed priming have been proven to be effective in promoting earlier planting [15]. While these technologies can alleviate crop phenological constraints, their inherent uncertainties hinder their widespread adoption.

Agricultural mechanization has the potential to alleviate phenological constraints [2]. Existing evidence suggests that agricultural mechanization, especially harvest mechanization, can facilitate more timely farm operations. This is essential to shorten the seasonal interval from harvesting to subsequent land preparation to enable multiple cropping [16]. For example, in comparison with manual harvesting, mechanized wheat harvest saves 94 hours per hectare [17]. This reduction in field operation time makes it possible to bring forward the planting date of the following crop, thereby extending the growing season length for the following crop [14]. However, there is still an absence of conclusive evidence on this issue, highlighting the need to investigate whether and how the harvest mechanization affects crop phenology in the multi-cropping system.

Winter wheat and summer corn double cropping, commonly known as wheat-corn double cropping, constitute one of the predominant cropping systems in China. Approximately 16 million hectares of arable land in China are dedicated to this rotation, contributing about 25% of the country’s total grain production [18]. However, the development of this system faces significant challenges due to seasonal constraints. Winter wheat is typically planted in late September or early October and harvested in the following June, after which summer corn is planted. This schedule results in both crops, especially summer corn, experiencing a shorter growing season. Consequently, in some regions, summer corn struggles to fully mature when planted at the usual time [19–21]. Efforts to advance the summer corn planting date have been limited by farmers’ prioritization of winter wheat [22]. Advancing the summer corn planting date often requires an earlier harvest of winter wheat, which could negatively affect its maturity and yield [23]. While intercropping—manually planting summer corn into wheat fields before the winter wheat matures—was previously used to solve this issue, it was eventually abandoned due to its labor-intensive nature and the susceptibility of seedlings to pests [24].

This study aims to investigate the spillover effect of mechanized harvest on crop phenology, drawing on the wheat-corn double cropping system in China as a case study. Specifically, we investigate the relationship between mechanized winter wheat harvest and the length of the subsequent summer corn growing season. These analyses are based on crop phenology data collected from agrometeorological monitoring stations that implemented the wheat-corn double cropping system in China from 1992 to 2013. The results show that the increased use of winter wheat harvest machinery remarkably extends the summer corn growing season. On average, a 167% increase in the intensity of winter wheat harvest machinery use results in an extension of the summer corn growing season by 13 days over the analysis period. Technically, this study uses the instrumental variable (IV) approach to address endogeneity issues associated with the use of winter wheat harvest machinery. We further demonstrate that the extended corn growing season, as investigated in this study, can be attributed to the advanced planting and delayed maturation of summer corn resulting from mechanized winter wheat harvest. Moreover, our examination of heterogeneity reveals that the impact of mechanized winter wheat harvest is more pronounced in regions with larger farm sizes and plain/hilly terrain.

This study contributes to the existing literature in two aspects. First, it is the first attempt to quantitatively investigate the relationship between mechanized winter wheat harvest and subsequent summer corn phenology in a wheat-corn double cropping system using granular county-level data. This finding of the spillover effect of mechanized winter wheat harvest in alleviating phenological constraints has the potential to facilitate double or triple cropping, thereby contributing to ensuring a stable grain supply in developing countries. Second, this study provides fundamental empirical evidence that mechanized winter wheat harvest affects the length of the summer corn growing season by enabling earlier planting and delaying the maturity of summer corn.

The subsequent sections of this study are structured as follows. The second section introduces a conceptual framework to elucidate the spillover impact of mechanized winter wheat harvest on the length of the summer corn growing season in a wheat-corn double cropping system. The third section outlines the empirical model and introduces the dataset utilized in the analysis. The fourth section presents the main results, followed by a discussion of their implications in the fifth section. The sixth section offers the study’s concluding remarks.

## 2. Conceptual framework

This section presents a conceptual framework regarding the key research question: how mechanized winter wheat harvest affects the length of the subsequent summer corn growing season in the wheat-corn double cropping system. While prior research has analyzed the crucial role of agricultural mechanization in enhancing land productivity [25], mitigating yield risk [26], increasing household income [27], as well as promoting gender-inclusive practices [28], there remains a gap in evidence regarding its impact on crop phenology to date.

Mechanized winter wheat harvest can potentially influence the summer corn growing season by advancing its planting date. Harvesting is one of the most labor-intensive tasks in crop production [29, 30], and a primary advantage of mechanized harvest is its ability to improve operational efficiency, significantly reducing the time required for field operations [31]. Studies by Kucharik [13] and Wang [14] have suggested that conservation tillage can narrow the operational window between the first crop’s harvest and the second crop’s planting, facilitating an earlier planting of the second crop in the same year. Similarly, efficient mechanized winter wheat harvest is expected to influence the planting date of the subsequent summer corn in the wheat-corn double cropping system by reducing the time required for winter wheat harvest, thereby shortening the time interval between winter wheat harvest and summer corn planting. While early planting is typically discouraged in wheat-corn double cropping systems due to competition for the growing season [20], the use of mechanized winter wheat harvest may enable this practice. Accordingly, we propose the first conjecture that mechanized winter wheat harvest would promote earlier planting of summer corn by shortening the time interval between winter wheat maturity and summer corn planting.

It is likely that mechanized winter wheat harvest would delay the summer corn maturity date. On the one hand, efficient mechanized winter wheat harvest leads to early planting of summer corn as a strategy to ensure the physiological maturity of summer corn before harvest [32]. On the other hand, early planting of summer corn encourages producers to select varieties characterized by longer growing seasons and greater yield potential. Prior studies have confirmed that corn yield with long-season hybrids exceeds that of short-season hybrids, given the appropriate planting date [33]. This conclusion suggests that rational farmers, seeking to maximize profits, have a strong intrinsic motivation to adopt long-season corn varieties with high yield potential when relatively sufficient growth periods are available. Changes in varieties would, in turn, affect the summer corn maturity date. In addition, warmer temperatures due to climate change provide preconditions for appropriately delaying the summer corn maturity date and the subsequent winter wheat planting date, all without decreasing the total output of these two crops [14]. Therefore, we propose the second conjecture that mechanized winter wheat harvest may extend the summer corn growing season by delaying the summer corn maturity date. This is a downstream effect of mechanized winter wheat harvest advancing the summer corn planting date.

**Fig 1:**
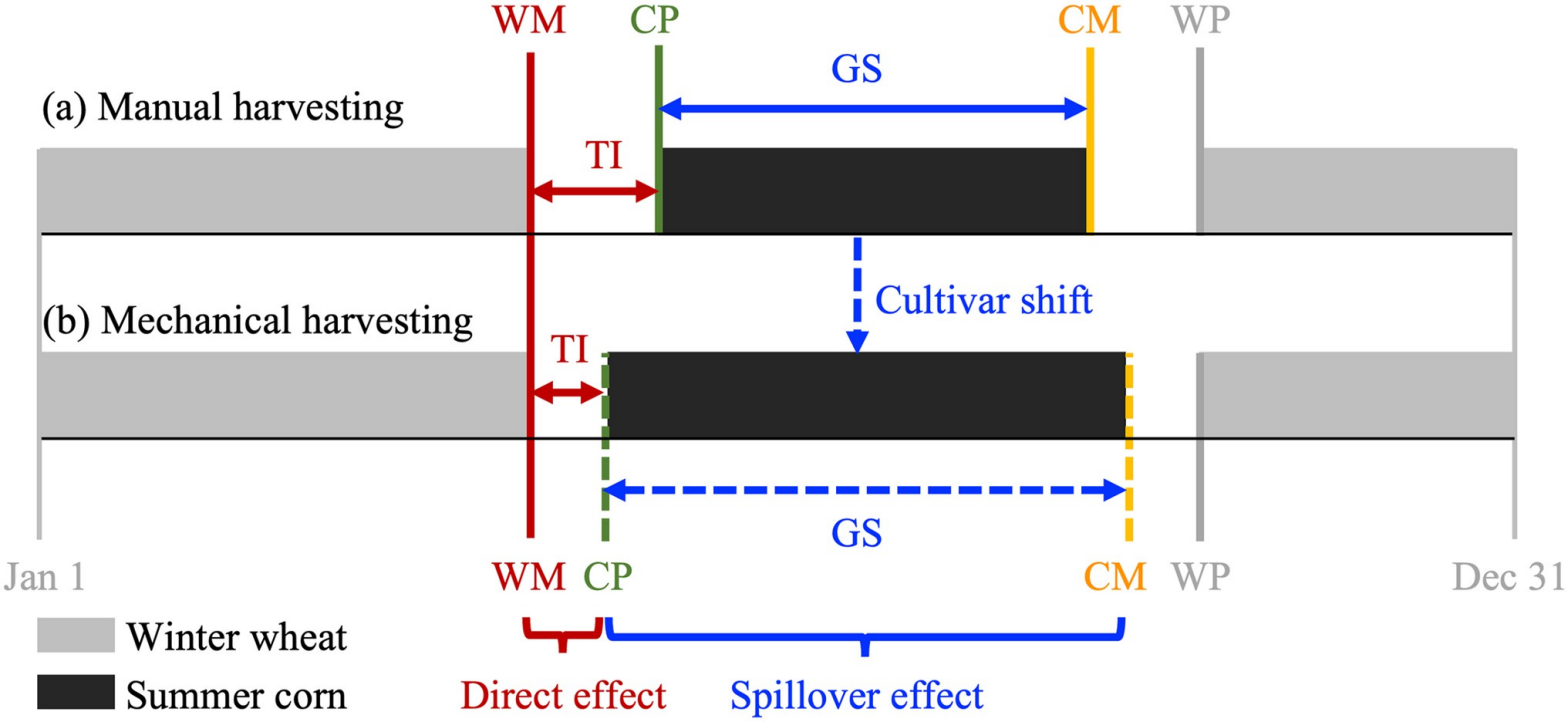
Schematic diagram illustrating the mechanism by which mechanized winter wheat harvest affects summer corn growing season length in a double cropping system. Notes: WM is the winter wheat maturity date, CP is the summer corn planting date, CM is the summer corn maturity date, WP is the winter wheat planting date, TI is the time interval between winter wheat maturity and summer corn planting, and GS is the length of the summer corn growing season.

Fig 1 illustrates the mechanism through which mechanized winter wheat harvest affects the length of the summer corn growing season in a double cropping system. This schematic diagram helps clarify the conceptual framework, which suggests that mechanized winter wheat harvest could extend the length of the summer corn growing season through two channels: advancing the planting date and delaying the maturing date of summer corn. Subsequently, we will use county-level crop phenology data to formally validate these conjectures.

## 3. Empirical strategy and data

### 3.1. Empirical strategy

#### 3.1.1. Baseline model specification

Building on the agronomic literature regarding the determinants of crop growing season length [34–38], and considering the limited but notable exceptions in the economic literature that provide causal estimates on how environmental changes affect the crop growing season [39], we propose the following model to investigate the spillover effect of mechanized winter wheat harvest on the length of the subsequent summer corn growing season:

$$Ln{GS}_{gt}=\alpha_{0}+\alpha_{1}\;\text{Ln}\;{HM}_{gt}+\alpha_{2}\;\text{Ln}\;{CM}_{gt}+\alpha_{3}{GDD}_{gt,l_{0}:\;l_{1}}^{GS}+\alpha_{4}{GDD}_{gt,l_{1}:\;l_{\infty }}^{GS}+\alpha_{5}P_{gt}^{GS}+T_{t}+\theta_{g}+\varepsilon_{gt}$$ (1)

where *GS*_*gt*_ represents the summer corn growing season length for county *g* in year *t*. The length of the summer corn growing season is defined as the number of days between the planting date and the maturity date, because the last stage of the summer corn growth cycle recorded by agrometeorological monitoring stations is maturity. Parameters ***α*** need to be estimated. *HM*_*gt*_ denotes the variable of interest, namely winter wheat harvest machinery use intensity. *CM*_*gt*_ represents machinery use intensity throughout the summer corn growing season. ${GDD}_{gt,l_{0}:\;l_{1}}^{GS}$ and ${GDD}_{gt,l_{1}:\;l_{\infty }}^{GS}$ denote the heat accumulated when the temperature is either at [*l*_0_°C, *l*_1_°C) or above *l*_1_°C, respectively, across the corn growing season. $P_{gt}^{GS}$ includes accumulated precipitation and its quadratic form during the summer corn growing season. *T*_*t*_ is the linear time trend to account for technological progress. County fixed effects (*θ*_*g*_) are incorporated to account for unobserved time-invariant factors such as land topography and soil quality. *ε*_*gt*_ is the error term.

This study uses growing-degree days (*GDD*), derived from a sine curve fit, to explore the nonlinear relationship between temperature and the length of the summer corn growing season [40]. The period for *GDD* computation is defined as May through October, because the planting and maturity of summer corn varies annually depending on local weather conditions. We employ a piecewise linear function to identify the nonlinear impacts of temperature [41]. Specifically, we establish the lower temperature threshold for summer corn at 10°C, then loop over all range of possible temperature thresholds from 20 to 40°C to determine the best fitting threshold for specific summer corn growing season lengths [42]. Ultimately, we select 34°C as the upper temperature bound (*l*_1_), implying that the nonlinear effect of temperature on the length of the summer corn growing season is captured by ${GDD}_{10-34{}^{\circ}C}^{GS}$ and ${GDD}_{34{}^{\circ}C+}^{GS}$.

However, due to potential endogeneity problems, estimating the coefficients of Eq (1) using ordinary least squares (OLS) regression may lead to biased estimates. Specifically, three sources of endogeneity could interfere with the identification of the causal impact of winter wheat harvest machinery on summer corn growing season length. First, reverse causality may exist between the length of the summer corn growing season and the use of winter wheat harvest machinery. A longer growing season, resulting in higher summer corn yields, may encourage the adoption of winter wheat harvest machinery. Second, the use of harvest machinery may suffer from a self-selection issue. Far from being random, this choice reflects an optimal choice by farmers under various constraints, such as the local labor market and terrain. The third source of endogeneity is the omitted variable problem. For example, agricultural practices that are challenging to measure, such as no-tillage and reduced-tillage, can affect crop phenology and may also be correlated with the use of agricultural machinery.

To address the potential endogeneity problems mentioned earlier, we employ the instrumental variable (IV) method. This method addresses unobserved confounders by exploiting “exogenous” sources of variation in the treatment [43, 44], which has become a prominent tool to make causal claims in the field of economics [45, 46]. Specifically, we use the average usage of winter wheat harvest machinery in neighboring counties in the previous year as an instrumental variable for current usage in the county of interest [26]. This practice is justified because a county’s harvest machinery usage tends to align closely with that of its neighbors, due to widespread cross-regional machinery services and similar agricultural machinery purchase subsidy policies in adjacent counties. However, the average one-year lagged usage of winter wheat harvest machinery in neighboring counties is unlikely to be correlated with the current summer corn growing season length in the county of interest. We estimate the causal impact of winter wheat harvest machinery on the length of the summer corn growing season using two-stage least squares (2SLS). The first-stage regression is expressed as follows:

$$\text{Ln}\;{HM}_{gt}=\beta_{0}+\beta_{1}\;\text{Ln}\;{HM}_{gt-1}^{N}+\beta_{2}\;\text{Ln}\;{CM}_{gt}+\beta_{3}{GDD}_{gt,l_{0}:\;l_{1}}^{GS}+\beta_{4}{GDD}_{gt,l_{1}:\;l_{\infty }}^{GS}+\beta_{5}P_{gt}^{GS}+T_{t}+\theta_{g}+\mu_{gt}$$ (2)

where ${HM}_{gt-1}^{N}$ denotes the average winter wheat harvest machinery use intensity in neighboring counties with a one-year lag. Parameters ***β*** are to be estimated, and *μ*_*gt*_ is an error term. Standard errors in the first and second stages are clustered at the county level and prefectural city-by-year level to account for serial correlation within counties over time and spatial correlation across counties within each city-year cell [47]. All other variables are defined as in Eq (1).

#### 3.1.2. Mechanism model specification

In this subsection, we establish models to explore the channels through which mechanized winter wheat harvest affects the length of the summer corn growing season. We begin by examining the impact of mechanized winter wheat harvest on the planting date of summer corn, and the regression model is specified as follows:

$$Ln{PD}_{gt}=\gamma_{0}+\gamma_{1}\;\text{Ln}\;{HM}_{gt}+\gamma_{2}\;\text{Ln}\;{CM}_{gt}+\gamma_{3}{GDD}_{gt,l_{0}^{\prime }:\;l_{1}^{\prime }}^{PD}+\gamma_{4}{GDD}_{gt,l_{1}^{\prime }:\;l_{\infty }^{\prime }}^{PD}+\gamma_{5}P_{gt}^{PD}+T_{t}+\theta_{g}+\vartheta_{gt}$$ (3)

where *PD*_*gt*_ represents the summer corn planting date for county *g* at year *t*. Since the summer corn planting date is strongly determined by the growth rate of winter wheat, and winter wheat usually enters a dormant stage in December, we use weather variables from December to June of the following year to explore the relationship between weather conditions and the summer corn planting date. We also use the piecewise linear function to assess the impacts of temperature, denoted by ${GDD}_{gt,l_{0}^{\prime }:\;l_{1}^{\prime }}^{PD}$ and ${GDD}_{gt,l_{1}^{\prime }:\;l_{\infty }^{\prime }}^{PD}$, setting the lower temperature bound ($l_{0}^{\prime }$) at 3°C [48]. We then loop all possible thresholds from 20°C to 35°C to determine the optimal upper temperature bound ($l_{1}^{\prime }$), finding that 26°C provides the best fit. $P_{gt}^{PD}$ includes accumulated precipitation and its quadratic form from December to the following June. Parameters ***γ*** are to be estimated, and *ϑ*_*gt*_ is an error term.

Before proceeding with this estimation, it is essential to first examine the direct effect of mechanized winter wheat harvest by examining its relationship with the time interval from winter wheat maturity to summer corn planting. Verifying this direct effect is a prerequisite for assessing any potential spillover effects of mechanized winter wheat harvest. To do so, we estimate a similar version of Eq (3), where the dependent variable (Ln*TI*_*gt*_) is the logarithmic form of the time interval between winter wheat maturity and summer corn planting. Some observations of the time interval variable (*TI*_*gt*_) are equal to zero, implying that summer corn was planted on the same day as winter wheat harvest. To ensure the logarithmic transformation is defined for all cases, we add a constant of 1 to each time interval before taking the natural logarithm, i.e., Ln(*TI*_*gt*_ + 1).

Another channel through which mechanized winter wheat harvest may affect the length of the summer corn growing season is by delaying the maturity date of summer corn. To further explore this relationship, we use the following regression specification:

$$Ln{MD}_{gt}=\delta_{0}+\delta_{1}\;\text{Ln}\;{HM}_{gt}+\delta_{2}\;\text{Ln}\;{CM}_{gt}+\delta_{3}{GDD}_{gt,l_{0}:\;l_{1}}^{MD}+\delta_{4}{GDD}_{gt,l_{1}:\;l_{\infty }}^{MD}+\delta_{5}P_{gt}^{MD}+T_{t}+\theta_{g}+\epsilon_{gt}$$ (4)

where *MD*_*gt*_ denotes summer corn maturity date in county *g* at year *t*. In this specification, we use the period from May to November to construct ${GDD}_{10-34{}^{\circ}C}^{MD}$ and ${GDD}_{34{}^{\circ}C+}^{MD}$, which capture the relationship between temperature and the summer corn maturity date. The model also includes accumulated precipitation from May to November and its quadratic term, represented by $P_{gt}^{MD}$. The parameters ***δ*** are to be estimated, and *ϵ*_*gt*_ represents the error term. Stata 16.0 was used to conduct all regression analyses.

### 3.2. Data

#### 3.2.1. Phenology data

Data on the phenology of winter wheat and summer corn from 1992 to 2013 are obtained from agrometeorological monitoring stations maintained by the China Meteorological Administration (CMA). These stations document specific dates for key growth stages of major crops, including planting, emergence, flowering, and maturity, across various regions of China. Phenological observations are conducted by adept agricultural technicians at each station following standardized observation criteria [49].

This study focuses on stations that have implemented a wheat-corn rotational system for a minimum duration of three years. In this system, winter wheat is typically planted in late September or early October and harvested in early June of the following year. Summer corn is then planted immediately after the wheat harvest and harvested in September [18, 50]. All crop phenology data used in the analysis are converted to days of the year (or DOY), representing the sequential count of days from January 1st.

**Fig 2:**
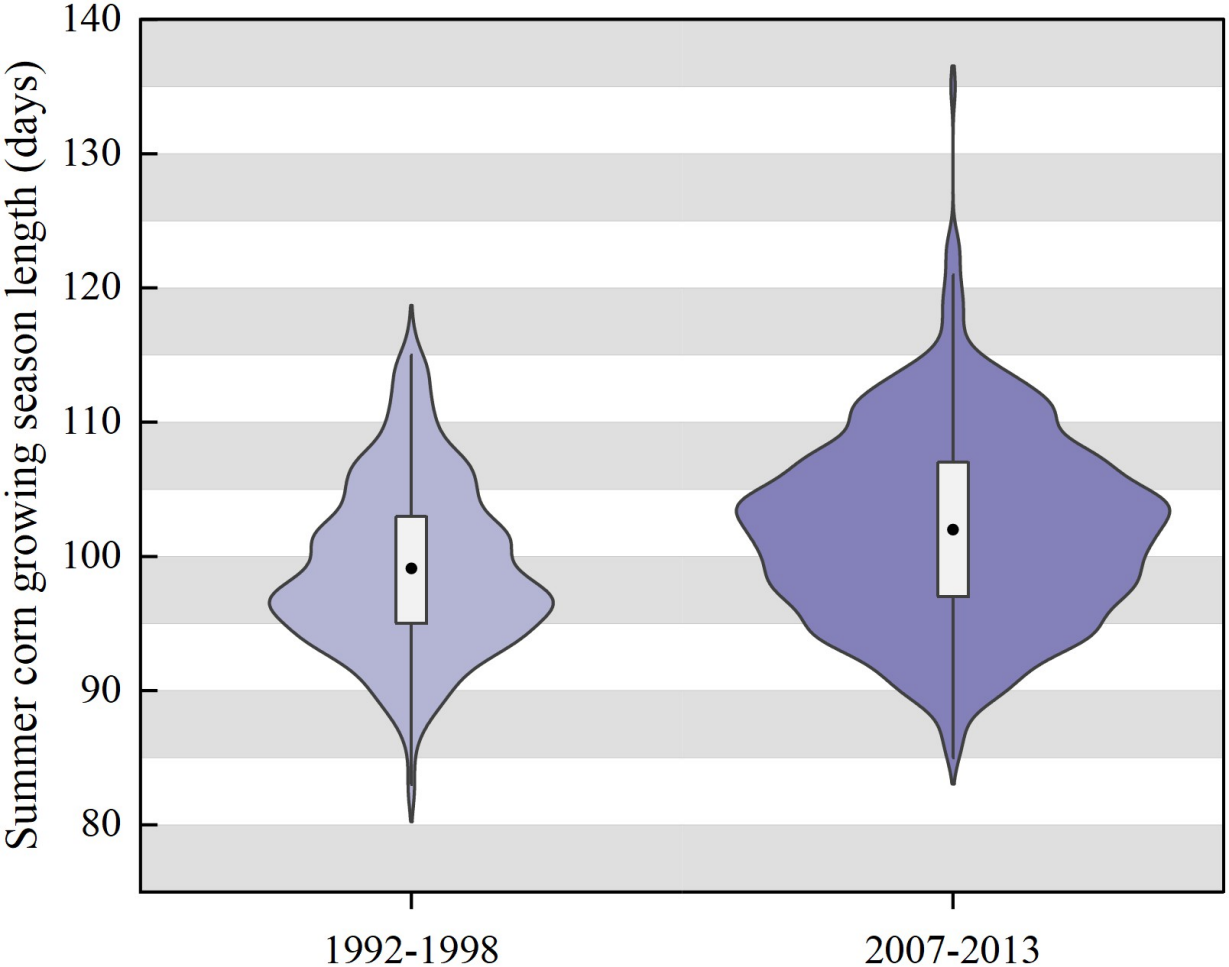
Changes in summer corn growing season length over time in China. Notes: The black solid circles in the middle of each violin plot represent the mean, while the upper and lower edges of the box represent the 75th and 25th percentiles, respectively.

Fig 2 depicts the length of the summer corn growing season in the wheat-corn double cropping system for the period 1992–2013. As is evident, the length of the summer corn growing season increased by an average of 3 days in the period 2007–2013 compared to the period 1992–1998. This change may be attributed to the enhanced efficiency of mechanized winter wheat harvest.

#### 3.2.2. Machinery data

Aggregated machinery use data at the county level for the period 1992–2013 are available from the Institute of Agricultural Information at the Chinese Academy of Agricultural Sciences (CAAS). Furthermore, data on crop-specific machinery use intensity at the provincial level for the same period can be obtained from the National Agricultural Product Cost-Benefit Data Compilation. However, direct data on machinery use intensity for specific crops at the county level is not available. To address this gap, we use the aforementioned machinery data to design a maximum entropy procedure (see S1 File in the Supporting Information).

Our results indicate that the discrepancy between the aggregated machinery usage observed annually at the county level and the total derived from the maximum entropy procedure is below 4.3% (results available from the author). As a result, we believe that the derived machinery usage data for wheat and corn are suitable for subsequent analysis. According to Xue [51] and Wang [26], winter wheat harvest machinery use is calculated by multiplying the total agricultural machinery use for winter wheat production by 0.22.

We integrate crop-specific machinery usage data with the crop phenology data to construct a county-level panel dataset. According to the method proposed by Arslan [52] and considering that the average area of counties with agrometeorological monitoring stations is about 2,247 km^2^, which closely corresponds to the area covered by a 30 km radius, we first establish a 30 km buffer around each agrometeorological monitoring station. Within these buffers, we identify the counties falling within their boundaries and assign the corresponding crop phenology information from the station to those counties. In cases where a county contains multiple agrometeorological monitoring stations within this range, we compute the average phenology dates across all stations. The final panel used in the analysis covers 340 counties across 10 provinces: Beijing, Tianjin, Hebei, Shandong, Jiangsu, Anhui, Henan, Shanxi, Shaanxi, and Xinjiang. Summary statistics for these variables are provided in Table 1.

**Table 1:** Summary statistics.

Variables	Mean	SD	Min	Max
Winter wheat maturity date (DOY)	157.448	6.924	135	178
Summer corn planting date (DOY)	164.556	7.061	141	187
Summer corn maturity date (DOY)	265.624	8.374	239	293
Winter wheat harvest machinery (kW/ha)	1.761	1.048	0.289	6.055
Summer corn machinery (kW/ha)	5.318	2.907	1.032	14.904
${GDD}_{10-34{}^{\circ}C}^{GS}$: from May to October (100D)	22.318	2.676	7.545	28.565
${GDD}_{34{}^{\circ}C+}^{GS}$: from May to October (100D)	0.043	0.049	0	0.500
${GDD}_{10-34{}^{\circ}C}^{MD}$: from May to November (100D)	22.664	2.783	7.545	29.253
${GDD}_{34{}^{\circ}C+}^{MD}$: from May to November (100D)	0.043	0.049	0	0.500
${GDD}_{3-26{}^{\circ}C}^{PD}$: from December to June (100D)	17.605	1.967	7.567	22.097
${GDD}_{26{}^{\circ}C+}^{PD}$: from December to June (100D)	0.655	0.294	0	1.489
Precipitation^GS^: from May to October (m)	0.485	0.193	0.005	1.443
Precipitation^MD^: from May to November (m)	0.504	0.200	0.005	1.481
Precipitation^PD^: from December to June (m)	0.180	0.083	0	0.671

Notes: “December to June” refers to the period from December to the following June. The number of observations is 5,554.

#### 3.2.3. Weather data

Data on weather for 820 stations across China, gathered at the station-day level, are obtained from the China Meteorological Data Sharing Service System. These data are converted from the station level to the county level using the inverse-distance weighting method, a commonly employed approach in the literature for spatially imputing weather data [53, 54]. Specifically, we spatially interpolate the weather data onto grids with a 500-meter grid spacing and then compute the average of all grids within each county to capture the county’s weather conditions.

## 4. Results

This section presents our empirical results. To avoid possible spurious regression caused by non-stationary time-series data, we begin by conducting unit root tests on all variables. Specifically, we use three panel unit root tests: the Im, Pesaran, and Shin (IPS) test [55], the Augmented Dickey–Fuller–Fisher (ADF–Fisher) test, and the Phillips–Perron–Fisher (PP–Fisher) test [56]. As detailed in S1 Table of the Supporting Information, all tests consistently reject the null hypothesis of a panel unit root at the 1% significance level, confirming that the variables are stationary.

Given that the RESET test (with a *p*-value greater than 0.1) rules out any functional form misspecification, we proceed with a Hausman test to ascertain whether the fixed-effects or random-effects model is more appropriate for estimating Eq (1). The results, with a *p*-value much less than 0.01, reject the random-effects model. Hence, we use the fixed-effects model to estimate the impact of mechanized winter wheat harvest on the length of the subsequent summer corn growing season.

Next, we perform a battery of robustness checks to validate the stability of the baseline results, followed by heterogeneity analyses to explore how the effects of mechanized winter wheat harvest on summer corn growing season length vary under different conditions. Last, we investigate the mechanisms at play by examining how mechanized winter wheat harvest affects the phenology of summer corn in the wheat-corn double cropping system.

### 4.1. Impact of mechanized wheat harvest on corn growing season length

#### 4.1.1. Baseline results

Table 2 presents the results of the 2SLS estimation, examining the impact of winter wheat harvest machinery on the length of the summer corn growing season. Columns (1) and (2) include the core explanatory variable, alongside controls for time trends and individual fixed effects. In Columns (3) to (6), additional control variables, such as weather conditions and other agricultural inputs, are progressively introduced to further refine the model.

**Table 2:** Effects of winter wheat harvest machinery on summer corn growing season length.

Variables	IV-2SLS estimation					
	First-stage	Second-stage	First-stage	Second-stage	First-stage	Second-stage
	(1)	(2)	(3)	(4)	(5)	(6)
Ln winter wheat harvest machinery		0.0750***		0.0681***		0.0755***
		(0.0111)		(0.0109)		(0.0128)
Ln lagged average winter wheat harvest machinery use in neighboring counties	0.6880***		0.6757***		0.6148***	
	(0.0333)		(0.0340)		(0.0312)	
Ln summer corn machinery					0.1311***	-0.0108
					(0.0373)	(0.0074)
${GDD}_{10-34{}^{\circ}C}^{GS}$			0.0109*	-0.0123***	0.0083	-0.0121***
			(0.0063)	(0.0016)	(0.0061)	(0.0016)
${GDD}_{34{}^{\circ}C+}^{GS}$			0.2356*	0.1277***	0.1410	0.1337***
			(0.1346)	(0.0419)	(0.1338)	(0.0423)
Precipitation^GS^			0.4123***	0.0379	0.5312***	0.0250
			(0.1122)	(0.0397)	(0.1202)	(0.0412)
Precipitation^GS^ squared			-0.1590*	-0.0300	-0.2106**	-0.0246
			(0.0851)	(0.0338)	(0.0897)	(0.0343)
Time trend	Yes	Yes	Yes	Yes	Yes	Yes
County-fixed effect	Yes	Yes	Yes	Yes	Yes	Yes
F-statistic	427.03***		394.90***		387.89***	
Partial R-squared	0.35		0.34		0.27	
Anderson-Rubin Wald test	52.60***		43.46***		37.53***	
Endogeneity test of endogenous regressors	23.35***		21.12***		20.17***	
Observations	5,554	5,554	5,554	5,554	5,554	5,554

Notes: In Columns (1), (3) and (5), the dependent variables are logarithmic form of the winter wheat harvest machinery. In Columns (2), (4) and (6), the dependent variables are logarithmic form of the summer corn growing season length. The standard errors in parentheses are clustered at county and city-by-year levels.

*** p<0.01,

** p<0.05,

* p<0.1.

Columns (1), (3) and (5) report the first-stage regression results, consistently demonstrating that the coefficients of the instrumental variable, namely average lagged winter wheat harvest machinery use in neighboring counties, are statistically significant at the 1% level. These results confirm the relevance of this instrument. The partial *R*-squared values, ranging between 0.27 and 0.35, further corroborate the strong predictive power of the instrument in relation to winter wheat harvest machinery [57].

Furthermore, the first-stage *F*-test statistics for the excluded instrument uniformly exceed the thresholds of 10 across all specifications [58, 59], reinforcing the validity of our instrumental variable. Similar support is provided by the Anderson-Rubin (AR) test, which remains robust even in the presence of weak instruments [60]. The *p*-values of AR Wald statistic, all below 0.01, strongly reject the null hypothesis that the coefficients of the endogenous variables are jointly equal to zero (i.e., the instrument is weak). These results provide compelling evidence that the average lagged winter wheat harvest machinery use in neighboring counties is a strong and valid instrument.

The 2SLS estimator, though generally less efficient than the OLS estimator when the explanatory variable (i.e., winter wheat harvest machinery) is exogenous, becomes necessary in the presence of endogeneity. Conducting an endogeneity test for this variable is useful to determine whether the application of the 2SLS estimation is warranted [61]. Accordingly, Table 2 reports the results of the endogeneity test, which is derived from the difference between two Sargan-Hansen statistics. This test is numerically equal to the Hausman test statistic but is robust to various violations of conditional homoskedasticity [62, 63]. As is evident, the results reject the null hypothesis that winter wheat harvest machinery is exogenous, at the 1% significance level. These findings support the treatment of winter wheat harvest machinery as an endogenous variable, suggesting that the IV method is more appropriate.

Columns (2), (4) and (6) of Table 2 present the second-stage estimation results. In all model specifications, the coefficients for winter wheat harvest machinery remain positive and statistically significant. These results confirm that mechanized winter wheat harvest extends the length of the subsequent summer corn growing season. Referring to Column (6), which is our preferred baseline model, a 1% increase in the intensity of winter wheat harvest machinery use leads to a 0.08% extension of the summer corn growing season.

We use the estimated coefficients to gain a more intuitive understanding of the magnitude of the change in summer corn growing season length due to increased winter wheat harvest machinery use from 1992 to 2013. First, we multiply the observed 167% increase in winter wheat harvest machinery use intensity over this period by the corresponding estimated coefficient in Column (6). This calculation yields the growth rate of summer corn growing season length attributable to mechanized winter wheat harvest. We then multiply this growth rate by the mean value of summer corn growing season length across the sample period. The result shows that the increased intensity of winter wheat harvest machinery use over the period 1992–2013 led to an extension of approximately 13 days in the summer corn growing season.

In addition, the nonlinear impact of temperature on the length of the summer corn growing season is captured by ${GDD}_{10-34{}^{\circ}C}^{GS}$ and ${GDD}_{34{}^{\circ}C+}^{GS}$. As shown in Column (6) of Table 2, *GDD* between 10 and 34°C shortens the length of the summer corn growing season, while *GDD* above 34°C leads to an extension of the summer corn growing season. This finding aligns with previous research conducted by Krishnan [64] and Parent and Tardieu [65], which suggests that elevated temperatures initially accelerate crop growth until a critical threshold is reached. Beyond this threshold, further increases in temperature slow the rate of crop development, resulting in a prolonged crop duration. However, we find that precipitation has an insignificant impact on the summer corn growing season length.

We also incorporate additional weather variables, including radiation and humidity, into Eq (1) to assess the sensitivity of our baseline results. As shown in Column (2) of S2 Table of the Supporting Information, the linear and quadratic terms for radiation and humidity are statistically insignificant. The coefficients for winter wheat harvest machinery closely resemble those from the baseline model. Hence, the exclusion of these additional weather variables does not affect the impact assessment of mechanized winter wheat harvest on the length of the summer corn growing season.

Furthermore, agricultural inputs such as labor, fertilizer, and irrigation may also have a minor impact on crop phenology [35, 37, 38]. To account for this, we incorporate these agricultural input variables into Eq (1) and report the regression results in Column (4) of S2 Table of the Supporting Information. The coefficient for winter wheat harvest machinery remains statistically significant and quantitatively similar to the baseline results reported in Column (6) of Table 2.

#### 4.1.2. Sensitivity analysis

Although we have tested the validity of the IV, there remains a possibility that unobserved variables could violate the assumptions of the IV method. To address this concern, we apply the “omitted variable bias” framework proposed by Cinelli and Hazlett [66] to perform a sensitivity analysis of our IV estimates. Specifically, we examine the strength of potential confounders required to bring the point estimate of IV (***α***_***IV***_) to zero. The IV estimator computed as the ratio of the reduced-form estimate (the effect of the instrument on the dependent variable) to the first-stage estimate (the effect of the instrument on the endogenous variable), expressed as ***α***_***IV***_ = ***α***_***RF***_**/*α***_***FS***_. Thus, our sensitivity analysis focuses on the reduced-form regression coefficient [66], which in this study involves examining the effect of the average lagged winter wheat harvest machinery use in neighboring counties on the length of the summer corn growing season.

**Fig 3:**
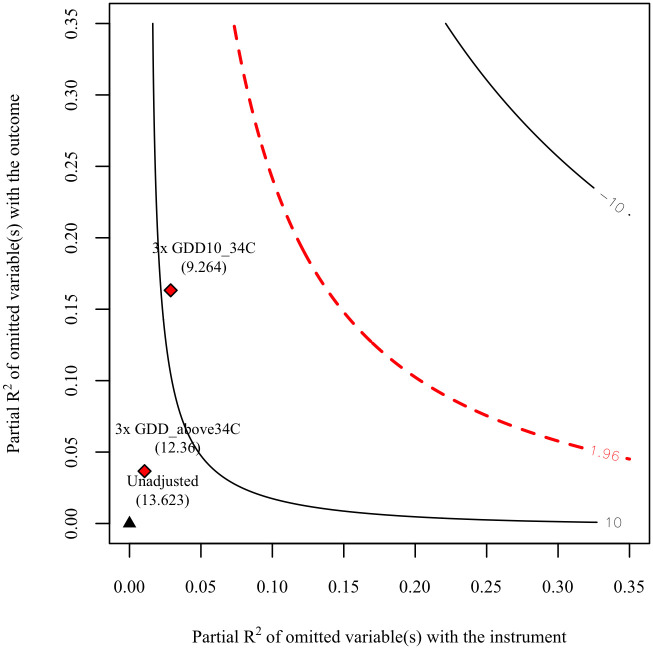
Sensitivity contour plots with benchmark bounds for the *t*-value of the reduced form. Notes: The contour lines represent the *t*-value that would result if an omitted variable of a given strength were incorporated into the reduced-form regression (i.e., the effect of the instrument on the dependent variable). The red dashed line indicates the threshold for statistical significance. Black triangles denote the case without omitted variables, while red diamonds represent the case with an omitted variable, whose strength is three times that of either ${GDD}_{10-34{}^{\circ}C}^{GS}$ or ${GDD}_{34{}^{\circ}C+}^{GS}$. The *t*-statistics for the corresponding models at these three points are reported in parentheses.

For this analysis, we use ${GDD}_{10-34{}^{\circ}C}^{GS}$ and ${GDD}_{34{}^{\circ}C+}^{GS}$ as the comparison benchmark for the omitted variables, given that temperature is undoubtedly an important variable affecting the length of the crop growing season [34, 36]. Fig 3 depicts the sensitivity contour plots, illustrating the benchmark bounds for the *t*-value. As we can see, even if the omitted variables are three times stronger than ${GDD}_{10-34{}^{\circ}C}^{GS}$ or ${GDD}_{34{}^{\circ}C+}^{GS}$, the null hypothesis of zero effect would still be rejected at the 5% significance level. This result enhances the reliability of our IV estimate.

Additionally, we undertake an array of checks to test the robustness of our baseline results. Specifically, we evaluate the sensitivity of our estimates to alternative buffers for identifying sample counties, different methods of measuring the dependent variable, varying periods for constructing weather variables, and alternative approaches to model spatial correlation. For brevity, the detailed results of these robustness checks are presented in the Supporting Information (S2 File). Overall, these results remain highly consistent with those of the baseline model.

#### 4.1.3. Heterogeneity analysis

To further understand the impact of mechanized winter wheat harvest on the length of the summer corn growing season under different topographic or socioeconomic conditions, this section performs a heterogeneity analysis. We first examine whether the impact of mechanized winter wheat harvest varies across the topographic conditions. Topographical relief plays a crucial role in the expansion of agricultural mechanization. Steep terrain not only hinders the accessibility of agricultural machinery in fields but also affects its operational efficiency [67]. Following the categories listed in the China Statistical Yearbook for the topographical conditions of the townships, we divide the sample into two groups: plains/hills and mountains. We expect the impact of winter wheat harvest machinery to be greater in plains/hills than in mountains. Columns (1)–(2) of Table 3 provide the results based on topographic conditions. We find a significantly positive coefficient for winter wheat harvest machinery in Column (1), while the coefficient is insignificant in Column (2). Not surprisingly, the rugged terrain of the mountainous areas hinders the adoption of winter wheat harvest machinery.

**Table 3:** Heterogeneity analysis.

Variables	Topographic conditions		Farm size	
	Plains & Hills	Mountains	Large	Small
	(1)	(2)	(3)	(4)
Ln winter wheat harvest machinery	0.0866***	0.0194	0.0953***	0.0616***
	(0.0160)	(0.0325)	(0.0257)	(0.0132)
Ln summer corn machinery	-0.0083	-0.0023	-0.0234**	-0.0010
	(0.0092)	(0.0209)	(0.0118)	(0.0078)
${GDD}_{10-34{}^{\circ}C}^{GS}$	-0.0120***	-0.0179***	-0.0156***	-0.0109***
	(0.0019)	(0.0035)	(0.0029)	(0.0015)
${GDD}_{34{}^{\circ}C+}^{GS}$	0.1328***	0.0859	0.1167*	0.1349***
	(0.0481)	(0.0987)	(0.0635)	(0.0441)
Precipitation^GS^	0.0104	0.0286	-0.0460	0.0624
	(0.0497)	(0.0584)	(0.0645)	(0.0406)
Precipitation^GS^ squared	-0.0188	-0.0137	0.0086	-0.0425
	(0.0414)	(0.0496)	(0.0548)	(0.0324)
Time trend	Yes	Yes	Yes	Yes
County-fixed effect	Yes	Yes	Yes	Yes
First-stage F-statistic	330.14***	111.46***	177.45***	274.87***
First-stage Partial R-squared	0.28	0.24	0.21	0.28
Anderson-Rubin Wald test	31.62***	0.37	15.59***	23.70***
Observations	3,372	660	2,008	3,546
Empirical *p*-values^†^	0.003***		0.005***	

Notes: This table reports the second stage results of 2SLS estimation. The dependent variables are logarithmic form of the summer corn growing season length.

^†^Fisher’s permutation test generates empirical *p*-values by executing 1000 repetitions of the bootstrap method, assuming the null hypothesis of coefficient equality in *Ln winter wheat harvest machinery* between the two samples. The group categorized by topographic conditions comprises 4,032 observations, as not all sample counties are covered in the classification found in the China Statistical Yearbook (Township). The standard errors in parentheses are clustered at county and city-by-year levels.

*** p<0.01,

** p<0.05,

* p<0.1.

We then explore whether the impact of mechanized winter wheat harvest on the length of the summer corn growing season varies with farm size. Mottaleb and Mohanty [68] suggest that agricultural machinery usage depends to a certain extent on the farm size, with larger farm sizes tending to use more agricultural machines. Here, we divide the sample into two groups based on the per-capita arable land in rural areas: the small-farm-size group and the large-farm-size group. The results, presented in Columns (3)–(4) of Table 3, indicate that winter wheat harvest machinery exhibits the anticipated sign and is statistically significant in both large- and small-farm-size groups. Following Cleary [69], we further use Fisher’s permutation test to test whether the coefficients for winter wheat harvest machinery differ significantly between these two groups. The resulting *p*-value is 0.005, indicating that the impacts of mechanized winter wheat harvest are more pronounced in regions with larger farm sizes.

### 4.2. Mechanism analysis

The evidence presented in Section 4.1 suggests that mechanized winter wheat harvest considerably extends the length of the subsequent summer corn growing season in a double cropping system. This section explores the mechanism behind this effect.

#### 4.2.1. Summer corn planting date

We use 2SLS estimation to identify the causal impact of mechanized winter wheat harvest on the summer corn planting date. Before conducting this estimation, we first examine the direct effect of mechanized winter wheat harvest on the time interval between winter wheat maturity and summer corn planting. Panel A of Table 4 provides the estimation results of this analysis. The results show that winter wheat harvest machinery has a significant negative impact on the time interval. Specifically, Column (1) shows that a 1% increment in intensity of the winter wheat harvest machinery use corresponds to a 0.43% reduction in the time interval. The 167% increase of winter wheat harvest machinery use intensity implies an approximate 5-day reduction in the time interval over the period of 1992–2013.

**Table 4:** Effects of winter wheat harvest machinery on the time interval and summer corn planting date using alternative weather measures.

Variables	Alternative measurement periods for weather variables			
	Dec.–Jun.	Sept.–Jun.	Jan.–Jun.	Feb.–Jun.
	(1)	(2)	(3)	(4)
**Panel A: Time interval between winter wheat maturity and summer corn planting (days)**				
Ln winter wheat harvest machinery	-0.4310**	-0.4229**	-0.4124**	-0.3989**
	(0.1976)	(0.1976)	(0.1961)	(0.1963)
First-stage F-statistic	261.14***	264.65***	260.03***	260.00***
First-stage Partial R-squared	0.23	0.24	0.24	0.23
Anderson-Rubin Wald test	4.78**	4.57**	4.43**	4.14**
**Panel B: Summer corn planting date (DOY)**				
Ln winter wheat harvest machinery	-0.0145*	-0.0176**	-0.0153**	-0.0147**
	(0.0075)	(0.0073)	(0.0074)	(0.0074)
First-stage F-statistic	379.68***	387.00***	382.32***	385.10***
First-stage Partial R-squared	0.28	0.28	0.28	0.28
Anderson-Rubin Wald test	3.75*	5.72**	4.28**	3.95**
Control variables	Yes	Yes	Yes	Yes
Time trend	Yes	Yes	Yes	Yes
County-fixed effect	Yes	Yes	Yes	Yes
Observations	5,554	5,554	5,554	5,554

Notes: This table reports the second stage results of 2SLS estimation. For Panel A, the dependent variables are logarithmic form of the time interval between winter wheat maturity and summer corn planting. For Panel B, the dependent variables are logarithmic form of the summer corn planting date. Control variables include summer corn machinery, ${GDD}_{3-26{}^{\circ}C}^{PD},\;{GDD}_{26{}^{\circ}C+}^{PD},\;{Precipitation}^{PD}$ and *Precipitation*^*PD*^ squared. The standard errors in parentheses are clustered at county and city-by-year levels.

*** p<0.01,

** p<0.05,

* p<0.1.

To test the robustness of the reported findings with respect to the measurement period of weather variables, we consider three alternative periods for measuring *GDD* and precipitation: September to the following June, January to June, and February to June. The results presented in Columns (2)–(4) of Panel A demonstrate that the conclusion—mechanized winter wheat harvest shortens the time interval between winter wheat maturity and summer corn planting—is robust.

Panel B of Table 4 presents the results of examining the impact of mechanized winter wheat harvest on the summer corn planting date using different periods for measuring weather variables. In each specification, the coefficient for winter wheat harvest machinery is consistently negative and statistically significant. To be specific, the estimates in Column (1) indicate that a 1% increase in the intensity of winter wheat harvest machinery use is associated with a 0.01% advancement in the summer corn planting date. This finding corresponds to an approximate 4-day earlier planting of summer corn from 1992 to 2013.

Overall, our results indicate that mechanized winter wheat harvest effectively shortens the time interval between winter wheat maturity and summer corn planting in the wheat-corn double cropping system, leading to an earlier planting date for summer corn. These findings validate our first research conjecture.

#### 4.2.2. Summer corn maturity date

We further investigate the impact of mechanized winter wheat harvest on the summer corn maturity date, with the results presented in Table 5. The estimated coefficient for winter wheat harvest machinery in Column (1) is positive and statistically significant at the 1% level. To be specific, each 1% increase in the intensity of winter wheat harvest machinery use corresponds to a 0.02% delay in the summer corn maturity date. Furthermore, we assess the cumulative effect of winter wheat harvest machinery use on the summer corn maturity date throughout the sample period. The results indicate that a 167% increase in the intensity of winter wheat harvest machinery use from 1992 to 2013 results in an approximate 9-day delay in the summer corn maturity date. As seen in Columns (2)–(4), the coefficients for winter wheat harvest machinery remain statistically significant and positive across the three alternative periods used for measuring weather variables. Overall, these findings support our second research conjecture, namely that mechanized winter wheat harvest extends the summer corn growing season by delaying the summer corn maturity date.

**Table 5:** Effects of winter wheat harvest machinery on the summer corn maturity date using alternative weather measures.

Variables	Alternative measurement periods for weather variables			
	May–Nov.	Jun.–Nov.	May–Oct.	Jun.–Oct.
	(1)	(2)	(3)	(4)
Ln winter wheat harvest machinery	0.0215***	0.0187***	0.0215***	0.0186***
	(0.0064)	(0.0064)	(0.0065)	(0.0064)
Ln summer corn machinery	-0.0011	0.0004	-0.0008	0.0008
	(0.0033)	(0.0033)	(0.0033)	(0.0033)
${GDD}_{10-34{}^{\circ}C}^{MD}$	-0.0065***	-0.0070***	-0.0068***	-0.0076***
	(0.0007)	(0.0007)	(0.0007)	(0.0008)
${GDD}_{34{}^{\circ}C+}^{MD}$	0.0613***	0.0829***	0.0614***	0.0849***
	(0.0179)	(0.0187)	(0.0177)	(0.0185)
Precipitation^MD^	0.0069	0.0388*	0.0024	0.0335
	(0.0227)	(0.0225)	(0.0221)	(0.0220)
Precipitation^MD^ squared	-0.0047	-0.0261	-0.0033	-0.0248
	(0.0202)	(0.0213)	(0.0204)	(0.0217)
Time trend	Yes	Yes	Yes	Yes
County-fixed effect	Yes	Yes	Yes	Yes
First-stage F-statistic	248.48***	246.22***	248.90***	246.71***
First-stage Partial R-squared	0.22	0.22	0.22	0.22
Anderson-Rubin Wald test	11.51***	8.86***	11.46***	8.76***
Observations	5,554	5,554	5,554	5,554

Notes: This table reports the second stage results of 2SLS estimation. The dependent variables are logarithmic form of the summer corn maturity date. The standard errors in parentheses are clustered at county and city-by-year levels.

*** p<0.01,

** p<0.05,

* p<0.1.

## 5. Discussion

The adoption of double- or triple-cropping systems is one of the most effective measures to ensure food security in China, particularly as the expansion of cropland becomes increasingly impractical and undesirable. However, the development of these systems faces significant challenges due to the shorter growing seasons associated with each crop. Extending the growing season is critical for boosting biomass accumulation and improving crop yields, as highlighted in previous studies [23, 37, 70, 71]. Despite this, the adoption of practices such as intercropping, which aim to extend crop growing seasons in multi-cropping systems, has been hindered by their inherent limitations [24].

Our results demonstrate that mechanized winter wheat harvest extends the subsequent summer corn growing season in a wheat-corn double cropping system. Over the period from 1992 to 2013, the intensity of winter wheat harvest machinery use increased by 167%, resulting in an average extension of the summer corn growing season by approximately 13 days. The evidence presented in this study indicates that mechanized winter wheat harvest could alleviate phenological constraints and may lead to an increase in summer corn yield in a double cropping system. This additional benefit of agricultural mechanization provides valuable insights for policy design aimed at ensuring food security, especially in contexts where arable land is scarce. To reap these benefits, policies should focus on factors that promote the development of agricultural mechanization, such as investing in innovative machinery technologies and improving rural road infrastructure. Additionally, raising farmers’ awareness of the benefits of using agricultural machinery and providing accessible credit services should also be considered as integral to agricultural mechanization efforts.

Our results also show that plain and hilly regions derive greater benefits from the use of winter wheat harvest machinery, indicating the importance of topographic conditions in advancing agricultural mechanization. By contrast, the rugged terrain of mountainous areas poses serious challenges to road accessibility and increases transportation costs, which may deter farmers from adopting mechanized practices [67]. Policies aimed at improving the suitability of agricultural machinery for mountainous areas would be particularly beneficial. In addition, our findings indicate that mechanized winter wheat harvest has a more pronounced impact on summer corn growing season length in regions with larger farm sizes, which is consistent with previous findings that expanding land scale through land transfer can help promote agricultural mechanization and achieve economies of scale [72, 73].

Our findings provide further evidence that mechanized winter wheat harvest extends the length of the summer corn growing season by reducing field operation time and advancing the planting date for summer corn. This finding aligns with the results of Mottaleb [74], which demonstrated that efficient farming machinery reduces turnaround time between crops. Furthermore, the downstream effect of mechanized winter wheat harvest on delaying the summer corn maturity date suggests a shift in corn cultivar choices. The earlier planting enabled by mechanized winter wheat harvest allows farmers to select corn varieties with longer growing seasons and higher yield potential, which to some extent explains the recent increases in corn production in China. As indicated by Liu [75], the adoption of longer-season corn cultivars has contributed to higher yields.

Moreover, it is important to acknowledge that this study has limitations. First, it is unable to inspect important aspects related to changes in summer corn cultivar with the data at hand. This gap remains to be further investigated using more granular data, such as data from the farm-level production surveys. Second, the benefits of using harvest machinery are not assessed in this study. In addition to the positive impact of mechanized winter wheat harvest on improving crop yield and reducing production cost for the first crop [31, 76], the spillover effects of mechanized winter wheat harvest on subsequent crop phenology could also influence the output of the second crop. Therefore, holistic approaches will be necessary in future studies to comprehensively evaluate the benefits of using harvest machinery in double cropping systems.

## 6. Conclusion

This study explores the relationship between mechanized harvest and crop phenology in a double cropping system. Using data on county-level crop phenology and agricultural machinery in China from 1992 to 2013, we examine the impact of mechanized winter wheat harvest on the length of the subsequent summer corn growing season in the wheat-corn double cropping system. Our findings indicate that mechanized winter wheat harvest extends the length of the summer corn growing season, alleviating the constraints imposed by its shorter growing period. The mechanism behind this is that mechanized winter wheat harvest advances the summer corn planting date by shortening the time interval between winter wheat maturity and summer corn planting, while also delaying the maturity date of summer corn. Additionally, we find that plains and hilly areas with larger farm sizes tend to benefit more from the use of winter wheat harvest machinery.

## Supporting information

S1 File Recover crop-specific machinery use intensity at the county level. (DOCX)

S2 File Robustness checks. (DOCX)

S3 File Data. (XLSX)

S1 Table Panel unit root test results. (DOCX)

S2 Table Effects of winter wheat harvest machinery on summer corn growing season length with additional control variables. (DOCX)

## References

[1] Pires GF, Abrahão GM, Brumatti LM, Oliveira LJC, Costa MH, Liddicoat S, et al. Increased climate risk in Brazilian double cropping agriculture systems: Implications for land use in Northern Brazil. Agric For Meteorol. 2016; 228–229:286–98.

[2] Wang F, He Z, Sayre K, Li S, Si J, Feng B, et al. Wheat cropping systems and technologies in China. Field Crop Res. 2009; 111(3):181–8.

[3] Kawasaki K. Two harvests are better than one: Double cropping as a strategy for climate change adaptation. Am J Agric Econ. 2018; 101(1):172–92.

[4] Seifert CA, Lobell DB. Response of double cropping suitability to climate change in the United States. Environ Res Lett. 2015; 10(2):024002.

[5] Shapiro BI, Brorsen BW, Doster DH. Adoption of double-cropping soybeans and wheat. J Agr Appl Econ. 1992; 24(2):33–40.

[6] Jiang Y, Yin X, Wang X, Zhang L, Lu Z, Lei Y, et al. Impacts of global warming on the cropping systems of China under technical improvements from 1961 to 2016. Agron J. 2021; 113(1):187–99.

[7] Ju H, van der Velde M, Lin E, Xiong W, Li Y. The impacts of climate change on agricultural production systems in China. Clim Change. 2013; 120(1–2):313–24.

[8] Kukal MS, Irmak S. U.S. Agro-climate in 20^th^ century: Growing degree days, first and last frost, growing season length, and impacts on crop yields. Sci Rep. 2018; 8(1):6977. 29725053 10.1038/s41598-018-25212-2 PMC5934404

[9] Prasad R, Gunn SK, Rotz CA, Karsten H, Roth G, Buda A, et al. Projected climate and agronomic implications for corn production in the Northeastern United States. PloS One. 2018; 13(6):e0198623. doi: 10.1371/journal.pone.0198623 29889853 PMC5995377

[10] Gornall J, Betts R, Burke E, Clark R, Camp J, Willett K, et al. Implications of climate change for agricultural productivity in the early twenty-first century. Philos Trans R Soc B. 2010; 365(1554):2973–89. doi: 10.1098/rstb.2010.0158 20713397 PMC2935125

[11] Kucharik CJ. A multidecadal trend of earlier corn planting in the central USA. Agron J. 2006; 98(6):1544–50.

[12] Sacks WJ, Deryng D, Foley JA, Ramankutty N. Crop planting dates: An analysis of global patterns. Global Ecol Biogeogr. 2010; 19:607–20.

[13] Kucharik CJ. Contribution of planting date trends to increased maize yields in the central United States. Agron J. 2008; 100(2):328–36.

[14] Wang J, Wang E, Yang X, Zhang F, Yin H. Increased yield potential of wheat-maize cropping system in the North China Plain by climate change adaptation. Clim Change. 2012; 113(3–4):825–40.

[15] Porter JR, Xie L, Challinor AJ, Cochrane K, Howden SM, Iqbal MM, et al. Food security and food production systems. In: Climate Change 2014: Impacts, Adaptation, and Vulnerability. Part A: Global and Sectoral Aspects. Contribution of Working Group II to the Fifth Assessment Report of the Intergovernmental Panel on Climate Change [Field, C.B., et al. (eds.)]. Cambridge University Press, Cambridge, United Kingdom and New York, NY, USA, 2014; pp. 485–533.

[16] Mrema GC, Baker D, Kahan D. Agricultural mechanization in sub-Saharan Africa: Time for a new look. Food and Agriculture Organization of the United Nations, Rome, 2008.

[17] Moussa A. Mechanical and traditional harvesting methods for wheat crop. Misr J Ag Eng. 2008; 25(4):1094–111.

[18] Yang X, Sun B, Zhang S. Trends of yield and soil fertility in a long-term wheat-maize system. J Integr Agr. 2014; 13(2):402–14.

[19] Lv Z, Li F, Lu G. Adjusting sowing date and cultivar shift improve maize adaption to climate change in China. Mitig Adapt Strat Gl. 2019; 25(1):87–106.

[20] Liang W, Carberry P, Wang G, Lü R, Lü H, Xia A. Quantifying the yield gap in wheat–maize cropping systems of the Hebei Plain, China. Field Crop Res. 2011; 124(2):180–5.

[21] Xiao D, Qi Y, Shen Y, Tao F, Moiwo JP, Liu J, et al. Impact of warming climate and cultivar change on maize phenology in the last three decades in North China Plain. Theor Appl Climatol. 2015; 124(3–4):653–61.

[22] Liu Z, Gao J, Gao F, Dong S, Liu P, Zhao B, et al. Integrated agronomic practices management improve yield and nitrogen balance in double cropping of winter wheat-summer maize. Field Crop Res. 2018; 221:196–206.

[23] Huang S, Lv L, Zhu J, Li Y, Tao H, Wang P. Extending growing period is limited to offsetting negative effects of climate changes on maize yield in the North China Plain. Field Crop Res. 2018; 215:66–73.

[24] Tao F, Zhang S, Zhang Z, Rotter RP. Maize growing duration was prolonged across China in the past three decades under the combined effects of temperature, agronomic management, and cultivar shift. Global Change Biol. 2014; 20(12):3686–99. doi: 10.1111/gcb.12684 25044728

[25] Ma W, Renwick A, Grafton Q. Farm machinery use, off-farm employment and farm performance in China. Australian J Agr Resource Econ. 2018; 62(2):279–98.

[26] Wang T, Yi F, Wu X, Liu H, Zhang YY. Calamitous weather, yield risk and mitigation effect of harvest mechanisation: Evidence from China’s winter wheat. Australian J Agr Resource Econ. 2024; 68(2):386–412.

[27] Jena PR, Tanti PC. Effect of farm machinery adoption on household income and food security: Evidence from a nationwide household survey in India. Front Sustai Food Syst. 2023; 7:922038.

[28] Fischer G, Wittich S, Malima G, Sikumba G, Lukuyu B, Ngunga D, et al. Gender and mechanization: Exploring the sustainability of mechanized forage chopping in Tanzania. J Rural Stud. 2018; 64:112–22.

[29] Pingali P. Agricultural mechanization: Adoption patterns and economic impact. Handb Agric Econ. 2007; 3:2779–805.

[30] Zhang X, Yang J, Thomas R. Mechanization outsourcing clusters and division of labor in Chinese agriculture. China Econ Rev. 2017; 43:184–95.

[31] Benin S. Impact of Ghana’s agricultural mechanization services center program. Agric Econ. 2015; 46(s1):103–17.

[32] Mo F, Sun M, Liu X, Wang J, Zhang X, Ma BL, et al. Phenological responses of spring wheat and maize to changes in crop management and rising temperatures from 1992 to 2013 across the Loess Plateau. Field Crop Res. 2016; 196:337–47.

[33] Sorensen I, Stone P, Rogers B. Effect of sowing time on yield of a short and a long season maize hybrid. Proc Agron Soc NZ. 2000; 30:63–6.

[34] Abbas G, Ahmad S, Ahmad A, Nasim W, Fatima Z, Hussain S, et al. Quantification the impacts of climate change and crop management on phenology of maize-based cropping system in Punjab, Pakistan. Agric For Meteorol. 2017; 247:42–55.

[35] Liu Y, Zhang J, Pan T, Ge Q. Assessing the adaptability of maize phenology to climate change: The role of anthropogenic-management practices. J Environ Manage. 2021; 293:112874. doi: 10.1016/j.jenvman.2021.112874 34058454

[36] He L, Asseng S, Zhao G, Wu D, Yang X, Zhuang W, et al. Impacts of recent climate warming, cultivar changes, and crop management on winter wheat phenology across the Loess Plateau of China. Agric For Meteorol. 2015; 200:135–43.

[37] Estrella N, Sparks TH, Menzel A. Trends and temperature response in the phenology of crops in Germany. Global Change Biol. 2007; 13(8):1737–47.

[38] Yi J, Li H, Zhao Y, Shao Ma, Zhang H, Liu M. Assessing soil water balance to optimize irrigation schedules of flood-irrigated maize fields with different cultivation histories in the arid region. Agr Water Manage. 2022; 265:107543.

[39] Cui X, Xie W. Adapting agriculture to climate change through growing season adjustments: Evidence from corn in China. Am J Agric Econ. 2021; 104(1):249–72.

[40] Baskerville GL, Emin P. Rapid estimation of heat accumulation from maximum and minimum temperatures. Ecology. 1969; 50(3):514–7.

[41] Kawasaki K, Uchida S. Quality matters more than quantity: Asymmetric temperature effects on crop yield and quality grade. Am J Agric Econ. 2016; 98(4):1195–209.

[42] Schlenker W, Roberts MJ. Nonlinear temperature effects indicate severe damages to U.S. Crop yields under climate change. Proc Natl Acad Sci. 2009; 106(37):15594–8. doi: 10.1073/pnas.0906865106 19717432 PMC2747166

[43] Angrist JD, Imbens GW, Rubin DB. Identification of causal effects using instrumental variables. J Amer Statistical Assoc. 1996; 91(434):444–55.

[44] Angrist JD, Pischke J-S. Mostly harmless econometrics: An empiricist’s companion. Princeton University Press, Princeton, 2009.

[45] Loayza NV, Raddatz C. The composition of growth matters for poverty alleviation. J Dev Econ. 2010; 93(1):137–51.

[46] Keane M, Neal T. Instrument strength in IV estimation and inference: A guide to theory and practice. J Econom. 2023; 235(2):1625–53.

[47] Cameron AC, Gelbach BJ, Miller LD. Robust inference with multiway clustering. J Bus Econ Stat. 2011; 29(2):238–49.

[48] Belyaeva M, Bokusheva R. Will climate change benefit or hurt Russian grain production? A statistical evidence from a panel approach. Clim Change. 2018; 149(2):205–17.

[49] CMA. Standard for agricultural climatological observations. China Meteorological Press, Beijing, 1993.

[50] Sun H, Zhang X, Chen S, Pei D, Liu C. Effects of harvest and sowing time on the performance of the rotation of winter wheat–summer maize in the North China Plain. Ind Crop Prod. 2007; 25(3):239–47.

[51] Xue C, Shi X, Zhou H. Influence path of agricultural mechanization on total factor productivity growth in planting industry. J Agrotechnical Econ. 2020; (10):87–102.

[52] Arslan A, Belotti F, Lipper L. Smallholder productivity and weather shocks: Adoption and impact of widely promoted agricultural practices in Tanzania. Food Pol. 2017; 69:68–81.

[53] Zhang P, Zhang J, Chen M. Economic impacts of climate change on agriculture: The importance of additional climatic variables other than temperature and precipitation. J Environ Econ Manage. 2017; 83:8–31.

[54] Deschênes O, Greenstone M. The economic impacts of climate change: Evidence from agricultural output and random fluctuations in weather: Reply. Am Econ Rev. 2012; 102(7):3761–73.

[55] Im KS, Pesaran MH, Shin Y. Testing for unit roots in heterogeneous panels. J Econom. 2003; 115(1):53–74.

[56] Phillips PCB, Perron P. Testing for a unit root in time series regression. Biometrika. 1988; 75:335–46.

[57] Sellars EA, Alix-Garcia J. Labor scarcity, land tenure, and historical legacy: Evidence from Mexico. J Dev Econ. 2018; 135:504–16.

[58] Stock JH, Watson MW. Introduction to econometrics (3rd updated edition). Pearson, 2015.

[59] Stock JH, Wright JH, Yogo M. A survey of weak instruments and weak identification in generalized method of moments. J Bus Econ Stat. 2002; 20(4):518–29.

[60] Anderson TW, Rubin H. Estimation of the parameters of a single equation in a complete system of stochastic equations. Annals of mathematical statistics. 1949; 20(1):46–63.

[61] Wooldridge JM. Introductory econometrics: A modern approach (5th ed.). Mason, Ohio: South-Western, Cengage Learning, 2013.

[62] Hayashi F. Econometrics. Princeton University Press, Princeton, New Jersey, 2000.

[63] Baum CF, Schaffer ME, Stillman S. IVREG2: Stata module for extended instrumental variables/2SLS and GMM estimation. Statistical Software Components S425401, Department of Economics, Boston College, 2002.

[64] Krishnan P, Ramakrishnan B, Reddy KR, Reddy VR. High-temperature effects on rice growth, yield, and grain quality. Adv Agron. 2011; 111:87–206.

[65] Parent B, Tardieu F. Temperature responses of developmental processes have not been affected by breeding in different ecological areas for 17 crop species. New Phytol. 2012; 194(3):760–74. doi: 10.1111/j.1469-8137.2012.04086.x 22390357

[66] Cinelli C, Hazlett C. An omitted variable bias framework for sensitivity analysis of instrumental variables. Available at SSRN 4217915. 2022.

[67] Ullah MW, Anad S. Current status, constraints and potentiality of agricultural mechanization in Fiji. AMA-Agr Mech Asia Afr Lat Am. 2007; 38(1):39–45.

[68] Mottaleb KA, Mohanty S. Farm size and profitability of rice farming under rising input costs. J Land Use Sci. 2015; 10(3):243–55.

[69] Cleary S. The relationship between firm investment and financial status. J Financ. 1999; 54(2):673–92.

[70] Mueller B, Hauser M, Iles C, Rimi RH, Zwiers FW, Wan H. Lengthening of the growing season in wheat and maize producing regions. Weather Clim Extreme. 2015; 9:47–56.

[71] Kamara AY, Ekeleme F, Chikoye D, Omoigui LO. Planting date and cultivar effects on grain yield in dryland corn production. Agron J. 2009; 101(1):91–8.

[72] Ma W, Zhu Z, Zhou X. Agricultural mechanization and cropland abandonment in rural China. Appl Econ Letters. 2021; 29(6):526–33.

[73] Li F, Feng S, Lu H, Qu F, D’Haese M. How do non-farm employment and agricultural mechanization impact on large-scale farming? A spatial panel data analysis from Jiangsu Province, China. Land Use Pol. 2021; 107:105517.

[74] Mottaleb KA, Krupnik TJ, Erenstein O. Factors associated with small-scale agricultural machinery adoption in Bangladesh: Census findings. J Rural Stud. 2016; 46:155–68. doi: 10.1016/j.jrurstud.2016.06.012 27524857 PMC4973803

[75] Liu Z, Hubbard KG, Lin X, Yang X. Negative effects of climate warming on maize yield are reversed by the changing of sowing date and cultivar selection in Northeast China. Global Change Biol. 2013; 19(11):3481–92. doi: 10.1111/gcb.12324 23857749

[76] Sims BG, Kienzle J. Farm power and mechanization for small farmers in sub-Saharan Africa. Agricultural and Food Engineering Technical Report NO.3. Food and Agriculture Organization of the United Nations, Rome, 2006.

