# Supplementary material for: Phenological responses of corn to agricultural mechanization: Evidence from a wheat-corn double cropping system in China
Source: PLoS One. 2024 Nov 1;19(11):e0312812. doi: 10.1371/journal.pone.0312812 (PMC11530014; doi:10.1371/journal.pone.0312812)
Supplement: S1 File — (DOCX) [file pone.0312812.s001.docx]

# Recover crop-specific machinery use intensity at the county level

This section briefly introduces the maximum entropy method. For a discrete distribution $p=(p_{1},\ldots, p_{K})$, Shannon’s information entropy is expressed as $-\sum_{k} p_{k}\ln p_{k}$, which is a measure of uncertainty. According to the Principle of Maximum Entropy [1], the solution maximizing entropy under known constraints can be described as the least biased constraint-satisfying distribution. Due to the convex nature of entropy optimization, there exists a unique solution. For an application of the Principle of Maximum Entropy in economic and econometric, see Golan [2].

Specifically, we use $A_{gi}$ to represent the planting area of crop $i$ in county $g$. Based on the reported statistics, we consider nine major types of crops, namely rice, wheat, corn, soybeans, potatoes, cotton, sugar crops, oil-bearing crops, vegetables and fruits. To estimate the machinery use intensity $M_{gi}$, we use $P_{gi}={S_{gi}}/S\in[0, 1]$ to represent the proportion of machinery use for crop $i$ in county $g$ to the total use in the corresponding province. The formulation of the maximum entropy problem is as follows:

|  | $\max_{P_{gi}} -\sum_{i=1}^{I} \sum_{g=1}^{G} P_{gi}\ln P_{gi}$, | (S.1) |
| --- | --- | --- |

subject to

|  | $\sum_{i=1}^{I} \sum_{g=1}^{G} P_{gi}=1$, | (S.2) |
| --- | --- | --- |
|  | $\sum_{i=1}^{I} P_{gi}=\frac{S_{g}}{S}$, $\forall g=1, 2, \ldots G$ | (S.3) |
|  | $M_{i}=\frac{\sum_{g=1}^{G} P_{gi}S}{\sum_{g=1}^{G} A_{gi}}$, $\forall i=1, 2, \ldots I$ | (S.4) |
|  | $M_{i}^{-}\frac{A_{gi}}{S}\leq P_{gi}\leq M_{i}^{+}\frac{A_{gi}}{S}$, $\forall g=1,2,\ldots,G \forall i=1, 2, \ldots I$ | (S.5) |

where $S_{g}$ represents the total machinery use in county $g$ reported by CAAS; $M_{i}$ denotes the provincial machinery use for each crop as reported in the National Agricultural Product Cost-Benefit Data Compilation; and $M_{i}^{-}$ and $M_{i}^{+}$represent the bounds for machinery use intensity.^[[1]](#footnote-1)^ Equation (S.2) is the normalization condition, Equation (S.3) satisfies the total use requirement at the county level, Equation (S.4) represents the provincial average machinery use intensity for each crop, and Equation (S.5) ensures that the machinery use intensity remains within a specific range. Given that $S_{gi}$ is a function of $P_{gi}$, the unknown county-level machinery use intensity $M_{gi}$ is also a function of $P_{gi}$. The maximum entropy method described above provides estimates of $P_{gi}$ for each crop and each county.

# References

1. Jaynes ET. Information theory and statistical mechanics. Phys Rev. 1957; 106(4):620-30.

2. Golan A, Judge GG, Miller D. Maximum entropy econometrics: Robust estimation with limited data. Chichester UK, Wiley, 1996.

1. The machinery use intensity displays considerable regional disparities in China due to differences in economic development and geographic conditions. For example, Yuhong district of Liaoning province recorded an intensity of 18 kW per hectare in 2012, whereas Liupan mountain region reported a markedly lower figure of 3.31 kW per hectare. Hence, we establish the bounds based on these observed regional differences in machinery use intensity within agricultural practices. [↑](#footnote-ref-1)
