# Supplementary material for: Phenological responses of corn to agricultural mechanization: Evidence from a wheat-corn double cropping system in China
Source: PLoS One. 2024 Nov 1;19(11):e0312812. doi: 10.1371/journal.pone.0312812 (PMC11530014; doi:10.1371/journal.pone.0312812)
Supplement: S2 File — (DOCX) [file pone.0312812.s002.docx]

# Robustness checks

In this section, we undertake an array of checks to test the robustness of our baseline results. First, we examine the robustness of our results to different buffer zones, which reflect the distance between the agrometeorological monitoring station and the sample counties. For the baseline model, we set a 30 km radius around each agrometeorological monitoring station to establish buffers and identify sample counties within these buffers. In further checks, we consider alternative radii below the 30 km threshold, including 5, 10, 15, 20, and 25 km to establish buffers and construct county-level crop phenology data. In S3 Table, we consistently detect significant positive impacts of mechanized winter wheat harvest across all columns, which confirms our previous findings.

Second, we verify the robustness of our baseline results by adopting an alternative approach to measure the summer corn growing season length. In our baseline model, we collect county-level summer corn phenology data by calculating the average date across all agrometeorological monitoring stations within each county. To address potential measurement error, we use an inverse distance weighted method to re-construct county-level crop phenology data. This method assigns weights based on the inverse of the distance between each agrometeorological monitoring station and the geographic center of the county. Stations located farther from the geographic center receive smaller weights in the calculations. The coefficient in Column (1) of S4 Table barely changes, indicating that the use of summer corn phenology data in the baseline model is not remarkably influenced by measurement error.

**S3 Table. Robustness checks: alternative buffers** **for sample counties identification.**

| **Variables** | **Alternative buffers** | | | | |
| --- | --- | --- | --- | --- | --- |
|  | **5 km** | **10 km** | **15 km** | **20 km** | **25 km** |
|  | **(1)** | **(2)** | **(3)** | **(4)** | **(5)** |
| Ln winter wheat harvest machinery | 0.0794*** | 0.0747*** | 0.0756*** | 0.0703*** | 0.0680*** |
|  | (0.0204) | (0.0167) | (0.0160) | (0.0144) | (0.0134) |
| Ln summer corn machinery | -0.0071 | -0.0039 | -0.0067 | -0.0058 | -0.0061 |
|  | (0.0114) | (0.0092) | (0.0087) | (0.0080) | (0.0074) |
| $\mathrm{GDD}_{10-34℃}^{\mathrm{GS}}$ | -0.0134*** | -0.0102*** | -0.0113*** | -0.0115*** | -0.0115*** |
|  | (0.0026) | (0.0021) | (0.0021) | (0.0018) | (0.0018) |
| $\mathrm{GDD}_{34℃+}^{\mathrm{GS}}$ | 0.1136** | 0.1287*** | 0.1396*** | 0.1340*** | 0.1385*** |
|  | (0.0543) | (0.0480) | (0.0455) | (0.0434) | (0.0427) |
| $\mathrm{Precipitation}^{\mathrm{GS}}$ | 0.0650 | 0.0636 | 0.0626 | 0.0436 | 0.0449 |
|  | (0.0465) | (0.0431) | (0.0435) | (0.0432) | (0.0415) |
| $\mathrm{Precipitation}^{\mathrm{GS}}$ squared | -0.0553 | -0.0426 | -0.0470 | -0.0327 | -0.0352 |
|  | (0.0350) | (0.0350) | (0.0361) | (0.0359) | (0.0342) |
| Time trend | Yes | Yes | Yes | Yes | Yes |
| County-fixed effect | Yes | Yes | Yes | Yes | Yes |
| First-stage F-statistic | 123.02*** | 205.08*** | 223.46*** | 293.43*** | 351.43*** |
| First-stage Partial R-squared | 0.22 | 0.24 | 0.24 | 0.24 | 0.26 |
| Anderson-Rubin Wald test | 16.52*** | 21.52*** | 25.21*** | 25.67*** | 26.97*** |
| Observations | 1,334 | 2,247 | 3,064 | 4,096 | 4,877 |

Notes: This table reports the second stage results of 2SLS estimation. The dependent variables are logarithmic form of the summer corn growing season length. The standard errors in parentheses are clustered at county and city-by-year levels. *** p<0.01, ** p<0.05, * p<0.1.

Third, we examine whether our results are sensitive to the different periods used for measuring weather variables. The period for constructing weather variables in the baseline estimates is specified as May through October. We further examine whether altering this period affects the estimated effects of mechanized winter wheat harvest, given that the actual growing period of summer corn may vary across regions due to uneven distribution of environmental endowments [1]. Specifically, we use three alternative periods: May to September, June to

**S4 Table. Robustness checks:** **alternative measures for dependent variables, weather periods, and spatial correlation.**

| **Variables** | **Distance-weighted** |  | **Alternative measurement periods**  **for weather variables** | | |  | **Spatial correlation** |
| --- | --- | --- | --- | --- | --- | --- | --- |
|  | **May – Oct.** |  | **May – Sept.** | **Jun. – Oct.** | **Jun. – Sept.** |  | **May – Oct.** |
|  | **(1)** |  | **(2)** | **(3)** | **(4)** |  | **(5)** |
| Ln winter wheat harvest machinery | 0.0762*** |  | 0.0755*** | 0.0700*** | 0.0664*** |  | 0.0755*** |
|  | (0.0129) |  | (0.0129) | (0.0123) | (0.0124) |  | (0.0131) |
| Ln summer corn machinery | -0.0109 |  | -0.0107 | -0.0085 | -0.0061 |  | -0.0108 |
|  | (0.0075) |  | (0.0073) | (0.0072) | (0.0071) |  | (0.0071) |
| $\mathrm{GDD}_{10-34℃}^{\mathrm{GS}}$ | -0.0120*** |  | -0.0134*** | -0.0154*** | -0.0195*** |  | -0.0121*** |
|  | (0.0017) |  | (0.0019) | (0.0018) | (0.0023) |  | (0.0016) |
| $\mathrm{GDD}_{34℃+}^{\mathrm{GS}}$ | 0.1336*** |  | 0.1391*** | 0.1855*** | 0.2152*** |  | 0.1337*** |
|  | (0.0427) |  | (0.0431) | (0.0421) | (0.0431) |  | (0.0423) |
| $\mathrm{Precipitation}^{\mathrm{GS}}$ | 0.0275 |  | 0.0320 | 0.0471 | 0.0601 |  | 0.0250 |
|  | (0.0416) |  | (0.0406) | (0.0395) | (0.0387) |  | (0.0394) |
| $\mathrm{Precipitation}^{\mathrm{GS}}$ squared | -0.0259 |  | -0.0348 | -0.0394 | -0.0529 |  | -0.0246 |
|  | (0.0345) |  | (0.0352) | (0.0359) | (0.0370) |  | (0.0324) |
| Time trend | Yes |  | Yes | Yes | Yes |  | Yes |
| County-fixed effect | Yes |  | Yes | Yes | Yes |  | Yes |
| First-stage F-statistic | 387.89*** |  | 384.97*** | 389.98*** | 385.54*** |  | 352.28*** |
| First-stage Partial R-squared | 0.27 |  | 0.27 | 0.27 | 0.27 |  | — |
| Anderson-Rubin Wald test | 37.46*** |  | 36.93*** | 34.08*** | 30.18*** |  | — |
| Observations | 5,554 |  | 5,554 | 5,554 | 5,554 |  | 5,554 |

Notes: This table reports the second stage results of 2SLS estimation. The dependent variables are logarithmic form of the summer corn growing season length. The standard errors in parentheses for Columns (1)–(4) are clustered at county and city-by-year levels. Conley spatial HAC standard errors in parentheses for Column (5) using the 200 km cutoff point. *** p<0.01, ** p<0.05, * p<0.1.

October, and June to September. Columns (2)–(4) of S4 Table report the results using alternative measurement periods for weather variables. Consistent with the baseline regression results, we find a significant and positive impact of winter wheat harvest machinery on the length of the summer corn growing season.

Fourth, we examine the robustness of the specification used to control for spatial correlation. Recognizing that adjacent counties in China may adopt similar production practice due to extensive cross-regional agricultural machinery services, accounting for spatial correlation becomes imperative. To address this, we use the procedure proposed by Colella [2] to calculate the Conley spatial HAC standard errors, which capture both serial and spatial correlations. Specifically, this approach allows for arbitrary correlation of standard errors among adjacent spatial observations, whereas the baseline model only considers spatial correlation between counties within a city for a given year. As shown in Column (5) of S4 Table, there is no change in the estimated coefficients of the variables, except for a slight change in the standard errors, which reinforces our previous results.

**References**

1. Cui X, Xie W. Adapting agriculture to climate change through growing season adjustments: Evidence from corn in China. Am J Agric Econ. 2021; 104(1):249-72.

2. Colella F, Lalive R, Sakalli SO, Thoenig M. Inference with arbitrary clustering. IZA Discussion Paper No. 12584, 2019.
