# Supplementary material for: Phenological responses of corn to agricultural mechanization: Evidence from a wheat-corn double cropping system in China
Source: PLoS One. 2024 Nov 1;19(11):e0312812. doi: 10.1371/journal.pone.0312812 (PMC11530014; doi:10.1371/journal.pone.0312812)
Supplement: S1 Table — (DOCX) [file pone.0312812.s004.docx]

**S1 Table.** **Panel unit root test results.**

| **Variables** | **IPS** | **ADF****–Fisher** | **PP–Fisher** |
| --- | --- | --- | --- |
|  | **(1)** | **(2)** | **(3)** |
| Ln summer corn growing season length | -15.2287*** | 1659.3971*** | 2051.6583*** |
| Ln time interval | -18.0602*** | 1765.5902*** | 2363.0930*** |
| Ln summer corn planting date | -16.7922*** | 1682.9602*** | 2072.9005*** |
| Ln summer corn maturity date | -19.8925*** | 1994.5819*** | 2536.1971*** |
| Ln winter wheat harvest machinery | -6.8716*** | 1061.1096*** | 1262.9082*** |
| Ln summer corn machinery | -11.5349*** | 1327.8258*** | 1790.9255*** |
| $\mathrm{GDD}_{10-34℃}^{\mathrm{GS}}$: from May to October | -14.4631*** | 1451.1986*** | 1636.3615*** |
| $\mathrm{GDD}_{34℃+}^{\mathrm{GS}}$: from May to October | -22.2367*** | 1983.0093*** | 3419.3093*** |
| $\mathrm{GDD}_{10-34℃}^{\mathrm{MD}}$: from May to November | -15.9621*** | 1543.0087*** | 1822.9851*** |
| $\mathrm{GDD}_{34℃+}^{\mathrm{MD}}$: from May to November | -22.2367*** | 1983.0093*** | 3419.3093*** |
| $\mathrm{GDD}_{3-26℃}^{\mathrm{PD}}$: from December to June | -16.5482*** | 1700.8288*** | 2365.7716*** |
| $\mathrm{GDD}_{26℃+}^{\mathrm{PD}}$: from December to June | -20.8416*** | 1934.5072*** | 3160.5331*** |
| $\mathrm{Precipitation}^{\mathrm{GS}}$: from May to October | -23.6014*** | 2309.8471*** | 4179.6830*** |
| $\mathrm{Precipitation}^{\mathrm{MD}}$: from May to November | -24.3313*** | 2328.1845*** | 5168.2749*** |
| $\mathrm{Precipitation}^{\mathrm{PD}}$: from December to June | -20.3336*** | 1956.2017*** | 2843.1586*** |

Notes: The time interval is the period from winter wheat maturity to summer corn planting. IPS denotes the Im, Pesaran and Shin W-stat; ADF**–**Fisher denotes the ADF**–**Fisher Chi-square; and PP**–**Fisher denotes the PP**–**Fisher Chi-square. The individual intercept and trend are included in the test equation, and the number of lags is selected based on the Akaike Information Criterion (AIC). *** p<0.01, ** p<0.05, * p<0.1.
