# Supplementary material for: Phenological responses of corn to agricultural mechanization: Evidence from a wheat-corn double cropping system in China
Source: PLoS One. 2024 Nov 1;19(11):e0312812. doi: 10.1371/journal.pone.0312812 (PMC11530014; doi:10.1371/journal.pone.0312812)
Supplement: S2 Table — (DOCX) [file pone.0312812.s005.docx]

**S****2 Table.** **Effects of winter wheat harvest machinery on summer corn growing season length** **with additional control variables.**

| **Variables** | **IV-2SLS estimation** | | | | |
| --- | --- | --- | --- | --- | --- |
|  | **First-stage** | **Second-stage** |  | **First-stage** | **Second-stage** |
|  | **(1)** | **(2)** |  | **(3)** | **(4)** |
| Ln winter wheat harvest machinery |  | 0.0737*** |  |  | 0.0890*** |
|  |  | (0.0134) |  |  | (0.0152) |
| Ln lagged average winter wheat harvest machinery use in neighboring counties | 0.5947*** |  |  | 0.5437*** |  |
|  | (0.0310) |  |  | (0.0342) |  |
| Ln summer corn machinery | 0.1353*** | -0.0091 |  | 0.0968*** | -0.0111 |
|  | (0.0386) | (0.0075) |  | (0.0333) | (0.0076) |
| $\mathrm{GDD}_{10-34℃}^{\mathrm{GS}}$ | 0.0185*** | -0.0123*** |  | 0.0085 | -0.0119*** |
|  | (0.0061) | (0.0017) |  | (0.0064) | (0.0017) |
| $\mathrm{GDD}_{34℃+}^{\mathrm{GS}}$ | 0.3874*** | 0.1247*** |  | 0.1535 | 0.1390*** |
|  | (0.1386) | (0.0439) |  | (0.1359) | (0.0434) |
| $\mathrm{Precipitation}^{\mathrm{GS}}$ | 0.2037* | 0.0416 |  | 0.4426*** | 0.0192 |
|  | (0.1234) | (0.0435) |  | (0.1166) | (0.0418) |
| $\mathrm{Precipitation}^{\mathrm{GS}}$ squared | -0.0504 | -0.0372 |  | -0.1537* | -0.0212 |
|  | (0.0900) | (0.0351) |  | (0.0880) | (0.0349) |
| $\mathrm{Radiation}^{\mathrm{GS}}$ | 0.0167 | -0.0179 |  |  |  |
|  | (0.0707) | (0.0220) |  |  |  |
| $\mathrm{Radiation}^{\mathrm{GS}}$ squared | -0.0042 | 0.0013 |  |  |  |
|  | (0.0056) | (0.0017) |  |  |  |
| $\mathrm{Humidity}^{\mathrm{GS}}$ | -0.0048 | -0.0040 |  |  |  |
|  | (0.0159) | (0.0045) |  |  |  |
| $\mathrm{Humidity}^{\mathrm{GS}}$ squared | 0.0001 | 0.0000 |  |  |  |
|  | (0.0001) | (0.0000) |  |  |  |
| Ln winter wheat harvest labor |  |  |  | -0.1542*** | 0.0366** |
|  |  |  |  | (0.0589) | (0.0163) |
| Ln summer corn labor |  |  |  | -0.0475 | 0.0116 |
|  |  |  |  | (0.0618) | (0.0160) |
| Ln summer corn fertilizer |  |  |  | 0.0306 | 0.0279** |
|  |  |  |  | (0.0480) | (0.0109) |
| Ratio of irrigated area |  |  |  | 0.1225*** | -0.0053 |
|  |  |  |  | (0.0423) | (0.0097) |
| Time trend | Yes | Yes |  | Yes | Yes |
| County-fixed effect | Yes | Yes |  | Yes | Yes |
| F-statistic | 368.69*** |  |  | 252.56*** |  |
| Partial R-squared | 0.25 |  |  | 0.20 |  |
| Anderson-Rubin Wald test | 31.50*** |  |  | 34.72*** |  |
| Observations | 5,554 | 5,554 |  | 5,398 | 5,398 |

Notes: The dependent variables are the logarithmic form of the summer corn growing season length. The ratio of irrigated area refers to the ratio of the practical irrigated area to the total planted area of summer corn. The number of observations in Columns (3)–(4) is reduced to 5,398 due to missing values in the ratio of irrigated area. Standard errors in parentheses are clustered at county and city-by-year levels. *** p<0.01, ** p<0.05, * p<0.1.
